# Effect of Phase Transformations on Scanning Strategy in WAAM Fabrication

**DOI:** 10.3390/ma14247871

**Published:** 2021-12-19

**Authors:** Muhammad Hassaan Ali, You Sung Han

**Affiliations:** Department of Mechatronics Engineering, Incheon National University, 119 Academy-ro, Yeonsu-gu, Incheon 22012, Korea; a.muhassaan@inu.ac.kr

**Keywords:** finite element analysis, phase transformation, transformation plasticity, wire arc additive manufacturing, the scanning pattern

## Abstract

Due to its high production rates and low cost as compared to other metal additive manufacturing processes, wire arc additive manufacturing (WAAM) has become an emerging technology in the manufacturing industry. However, the residual stress generation and part distortion hinder its widespread adoption because of the complex thermal build-histories of WAAM parts. One of the ways to alleviate this problem is to consider the effects of scan strategies as it directly influences the thermal history of the built part. Since WAAM itself is an evolved welding process and even though it is evident from welding studies that phase transformations directly affect the residual stresses in welded parts, it remains unclear how the consideration of phase transformations for different scan strategies will affect the residual stresses and distortions in the WAAMed parts. A FEM study has been performed to elucidate the effects of phase transformations on residual stresses and the distortion for different deposition patterns. The current findings highlight that for the fabrication of low-carbon martensitic steels: The consideration of phase transformations for line-type discontinuous patterns (alternate and raster) do not significantly affect the residual stresses. Consideration of phase transformations significantly affects residual stresses for continuous patterns (zigzag, in–out and out–in). To accurately simulate complex patterns, phase transformations should be considered because the patterns directly influence the temperature history of the built part and will thus affect the phase transformations, the residual stresses and the warpage. During the fabrication of WAAM parts, whenever possible, discontinuous line scanning patterns should be considered as they provide the part with uniform residual stress and distortion. The alternate line pattern has been found to be the most consistent overall pattern.

## 1. Introduction

Additive manufacturing (AM) has recently garnered a lot of interest because it allows for the production of complex geometrical parts, fast prototyping, and a significant reduction of maintenance and repair costs. In the AM process, a component is produced via layer-by-layer deposition. The ability of AM to produce near-net-shape parts makes it an attractive process for short production runs.

The metal AM process can be categorized either by direct metal/energy deposition (DMD/DED) or by powder bed fusion (PBF). In the DED process, a heat source is applied to the solid material for the deposition, while in PBF, material which is in the form of metallic powders is spread onto a powder bed and the heat source fuses the powders in each layer. In both processes, melting is involved by a focused heat source such as a laser, arc, or electron beam. 

Wire arc additive manufacturing (WAAM) is a wire-feeding DED process. The WAAM product is built up through the deposition of welding beads. In WAAM, the material experiences complex multi-physics phenomena due to severe thermal inhomogeneities over multiple passes and layer. These complex thermal histories induce complex microstructural changes due to phase transformations and alter the mechanical properties of the built part. Each phase transformation plays as a source of deformation due to the volume difference among the coexisting phases and the transformation plasticity [1]. 

As a process itself, WAAM is inherently similar to welding, because it is essentially derived from welding and WAAM machines can even be retrofitted from arc welding robots [2]. Thus, process parameters (such as heat source power, size, and speed) that affect welding also significantly affect these AM processes. There are additional parameters particular to AM which also need to be considered such as the scan pattern, the hatch size spacing, powder feed rate, cooling time between layers, ambient temperature conditions, etc. to name a few. The thermal histories of the manufactured parts are imperative in determining the resulting microstructure, part distortion, and residual stresses. Explicitly, part distortion and residual stresses may cause failure in the form of buckling, fatigue, brittle fracture, and stress-induced cracking. 

Li et al. [3] performed a numerical study to examine the effect of AM scanning patterns on residual stress in the Aluminum alloy 2319. They investigated several representative scan patterns such as zig-zag, raster, alternate, in–out spiral, and out–in spiral. Cheng et al. [4] have compared the scan patterns for selective laser melting in In718. They concluded that 45° inclined line scanning patterns resulted in reduced residual stresses than other cases considered. Jia et al. [5] conducted both numerical and experimental studies on the effects of scanning patterns on selective laser melted parts of Ti-6Al-4V. They found that a 15° rotated scan strategy generated the smallest level of residual stresses in their study. Somashekara et al. [6] studied the effects of scan patterns on residual stresses on steel twin-wire welding-based additive manufacturing (TWAM). While the results show that the raster patterns are recommended for TWAM manufacturing, the authors emphasize that the accuracy of the results for the numerical model can be improved by the consideration of phase transformations. 

While much literature exists on phase transformation on welded parts as discussed in this review [7], limited implementations exist for AM products. For example, Bailey et al. [8] considered the phase transformations in steel for a multi-layer and multi-track laser direct deposition (LDD) to find the variations in hardness and stresses along with the depth and verified it with experimental results. They found that the high fractions of matensite in the heat-affected zones result in strong compressive stresses and that the multiple laser passes alleviate those compressive stresses to some extent. They also found that the ultimate tensile strength of H13 steel was 15 to 30% higher than the reported values of commercial H13. Xavier et al. [9] performed experimentally informed simulations on the effects of phase transformation on residual stress of WAAMed B91 steel sample with the employment of the modified Koistinen–Marburger formula. Xavier pointed out that for WAAM of martensitic steels phase transformations play a major effect on the residual stresses of the final part, especially in the area near the deposits.

The AM scanning strategy has significant effects on the deformation and the residual stress distribution of the final parts. A proper deposition path can reduce residual stress and deformation, especially in large-scale WAAM manufacturing. In the present study, a finite element study has been performed to investigate the effects of scanning strategy on residual stress and distortion in WAAM fabrication with an account of phase transformation and transformation plasticity in EH36 steel. A systematic finite element model with hypoelastic formulation based on additive decomposition has been employed. The residual stress and the distortion of WAAM part with and without considering phase transformation are compared and investigated for different AM scanning strategies. The objective of the present study is to signify the effects of considering phase transformations for different AM scan patterns and its effects on the final residual stresses and distortions on the built parts.

## 2. Methodology

### 2.1. FEA Model

EH36 is a widely used material in the shipbuilding industry due to its high strength, excellent welding performance, and good toughness at low temperature. In this study, EH36 steel has been selected for both the substrate and the wire deposition in WAAM. Such selection has been made for a potential application of AM in the maritime and shipbuilding industry. The chemical composition of EH36 (in wt.%) is 0.14% C, 1.47% Mn, 0.28% Si, 0.05% Cr, 0.3% Ni, 0.01% Mo, 0.002% S, 0.04% Al, 0.03% V, 0.03% Nb, 0.2% Cu, and 0.02% P and Fe balance.

**Figure 1:**
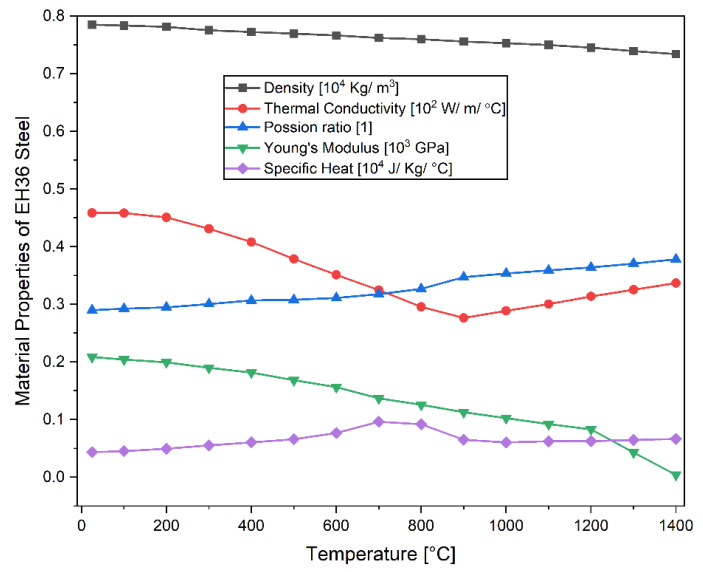
Temperature-dependent material properties of EH36 steel, adapted from [10].

Figure 1 shows the temperature-dependent material properties used in this study [10]. The values of the latent heat (251,400 J/Kg) and the solidus (1465.1 °C) and the liquidus (1522.5 °C) temperatures have been taken from [11]; The mechanical properties of the yield stress, the thermal strain, and the hardening coefficient in [1] are employed for the present study. The effects of meltpool dynamics has been ignored in the present study.

**Figure 2:**
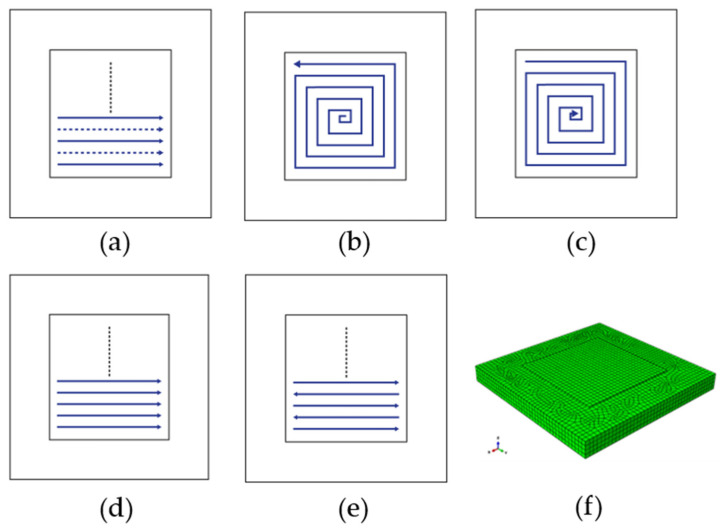
Deposition patterns analyzed ((**a**) alternate, (**b**) in–out, (**c**) out–in, (**d**) raster, and (**e**) zigzag) and (**f**) the FE model used in the present work.

In this work, five scanning patterns are examined such as alternate, in–out spiral, out–in spiral, raster, and zigzag as shown in Figure 2a–e. The details on the deposition pattern have been provided in the next section. The substrate has dimensions of 200 mm × 200 mm × 20 mm and a single deposition layer of 120 mm × 120 mm × 2.3 mm as can be seen in Figure 2f. The geometry contains a total of 7672 elements of which, the deposition layer has 576 elements and the substrate has a total of 7096 elements. A sequentially coupled thermo-mechanical analysis has been performed in this study while keeping the other WAAM parameters such as the welding speed, power and, geometry of the heat source constant. Element for the thermal simulations are DC3D8, 8-node linear heat transfer brick elements, whereas for the mechanical simulations C3D8, 8-node linear brick elements were used.

The WAAM process was simulated using the AM-plugin in ABAQUS 2019( Dassault Systèmes, Vélizy-Villacoublay, France), in which the analysis deploys the progressive element activation for the material deposition. In the previous study, the author implemented the finite element codes for the welding simulations with consideration of phase transformation and transformation plasticity [1] by way of ABAQUS user sub-routine DFLUX, UMAT, and UEXPAN. Such ABAQUS user sub-routine codes are used along with AM-plug-in ABAQUS 2019 in the present study. In the previous work, the DFLUX was used in ABAQUS to define the position, velocity, and volumetric heat flux of the heat source, whereas in this study this function has been performed by using the AM-plug-in. The UMAT is implemented to material’s mechanical behavior in the elastoplastic constitutive equations using a hypoelasticity based formulation, while the UEXPAN code provides the thermo-metallurgical strains considering the phase transformations. Further details regarding the implementation of the model and the calibration of the continuous cool transformation (CCT) curves have been provided in the methods section. 

### 2.2. Deposition Patterns

Residual stress and part deformation of AM products are largely influenced by their thermal histories such as temperature distribution and cooling rate. In the AM process, the localized heat input can cause a significant temperature gradient across the AM products. Such gradient, in turn, contributes to varying stresses throughout the AM product. In this work, the focus is on the influence of AM scan pattern on phase transformation evolution by thermal history changes, and eventually on the residual stresses and the warpage of the final products. Five representative scan patterns are analyzed, which are alternate, in–out, out–in, raster, and zigzag as shown in Figure 2a–e. Alternate, raster, and zigzag are line-path patterns, while in–out and out–in are contour-path patterns. Both the alternate and the raster are discontinuous patterns because they need multiple starts and stops during the whole deposition process. The zigzag is a continuous line pattern, which does not require multiple starts and stops and thus reduces the number of passes. However, this poses the requirement to precisely control the process parameters [12]. The in–out and the out–in patterns follow spiral contours and allow for fewer passes. However, they require a large number of turns and produce excessive thermal gradients at the beginning and the end of the scan, respectively. Such excessive heat input can lead to the formation of voids [13]. 

### 2.3. Thermal Analysis

The transient heat conduction equation has been utilized to model the heat transfer in this work as follows:

(1) $$C_{p}\rho \frac{dT}{dt}=\frac{\partial }{\partial x}\left(k\frac{\partial T}{\partial x}\right)+\frac{\partial }{\partial y}\left(k\frac{\partial T}{\partial y}\right)+\frac{\partial }{\partial z}\left(k\frac{\partial T}{\partial z}\right)+Q$$

where $C_{p}$, ρ, and k are the heat capacity, density, and thermal conductivity, respectively; T is the temperature and Q is the input heat source term.

The double ellipsoidal heat source model proposed by Goldak et al. [14] is used to simulate the heat input in AM. The power density equations, for the front and rear parts, are given as follows:

(2) $$Q_{f}=\frac{6\sqrt{3}f_{f}Q}{a_{f}bc\pi \sqrt{\pi }}\exp\left(-\frac{3x^{2}}{a_{f}^{2}}-\frac{3y^{2}}{b^{2}}-\frac{3z^{2}}{c^{2}}\right)$$

(3) $$Q_{r}=\frac{6\sqrt{3}f_{r}Q}{a_{r}bc\pi \sqrt{\pi }}\exp\left(-\frac{3x^{2}}{a_{r}^{2}}-\frac{3y^{2}}{b^{2}}-\frac{3z^{2}}{c^{2}}\right)$$

where *x*, *y*, and *z* are the spatial coordinates of the heat source, which follow the deposition path; $a_{f}$ and $a_{r}$ represent the front and rear lengths of the ellipsoid, respectively; b and c represent the width and depth of the heat source, respectively. *Q* is the amount of energy input. The choice of $f_{f}+f_{r}=2$ has been made according to [15]. The values of the parameters used in the present study can be found in Table 1. They were matched and calibrated based on the works in [15,16]. Convection and radiation boundary conditions that have been applied for the deposited layer are 5.7 and 0.2 W/m^2^K, respectively [15]. For the substrate, an equivalent convection coefficient for heat loss was imposed. The value of 167 W/m^2^K was found to be the most suitable to match the temperature profile of the heat source to Ding’s work [15]. Other considerations were made by utilizing the power-speed correlations from Hu and Qin’s work [17]. The scanning speed is set to be 10 mm/s.

### 2.4. Metallurgical Analysis

In AM processes, cooling rates across the part may vary depending on the type of the scan pattern. Such different cooling rates result in different rates of carbon diffusion and thus different phase volume fraction of the final product. The residual stresses after AM process, are attributed to two different sources as follows:

1. Plastic deformation on the macroscopic scale due to large temperature gradients.
2. Transformation plasticity on a microscopic scale due to solid-state phase transformations and corresponding volumetric dilatation.

In order to take into account such effects, in the present study, the phase evolution equation proposed by Leblond et al. [18] is coupled with the energy equation and implemented to the numerical model as follows:

(4) $${\dot{p}}_{i}=-\sum_{j=1,j\neq i}^{N}A_{ij}\left(T,\dot{T}\right),\;i=1,2,\ldots ,N$$

(5) $$\sum_{i=1}^{N}p_{i}=1\;\mathrm{for}\;t>0$$

In Equation (4), *p* is the volume fraction of each phases in consideration; *N* is the total number of phases, which is set to be 4 (e.g., *i* = 1, 2, 3, and 4 represents ferrite-pearlite, bainite, martensite, and austenite, respectively). $A_{ij}\left(T,\dot{T}\right)$, refers to the rate of phase transformed from *j* to *i*; $A_{ij}\left(T,\dot{T}\right)$, is explained with more detail in Section 2.4.1.

For martensitic transformation, the Koistinen–Marburger model [19] is employed as follows:

(6) $$\begin{array}{c} p_{3}\left(T\right)=1-\exp\left\{a\left(T_{3,S}-T\right)\right\}\left(T\leq T_{3,S}\right)\\ \;\mathrm{with}\;a=-\frac{\ln\left[p_{1}\left(T_{3,S}\right)+p_{2}\left(T_{3,S}\right)\right]}{T_{3,S}-T_{3,F}} \end{array}$$

Here, *T* is the current temperature and, $T_{3,S}$ and $T_{3,F}$ are the start and finish temperatures for the martensitic transformation. The energy equation with an account of phase transformation and the thermal boundary conditions are incorporated together as in Equations (7)–(9). Once each phase volume fractions are calculated by integration of Equation (4) using the trapezoidal rule, such results are plugged into Equation (7) for thermal analysis.

(7) $$\sum_{i}p_{i}{\left(\rho c\right)}_{i}\frac{dT}{dt}+\sum_{i}{\dot{p}}_{i}\rho_{i}H_{i}=\nabla \cdot \left(\sum_{i}p_{i}\lambda_{i}\nabla T\right)$$

(8) $$\left(-\right)\lambda \frac{\partial T}{\partial n}=q\;\mathrm{on}\;S_{q}$$

(9) $$\left(-\right)\lambda \frac{\partial T}{\partial n}=\chi_{1}\left(T-T_{0}\right)+\chi_{2}\left(T^{4}-T_{0}^{4}\right)\;\mathrm{on}\;S_{\theta }$$

$\rho_{i},\;c_{i},\;H_{i},\;\lambda_{i}$ are the density, specific heat capacity, enthalpy, and thermal conductivity of phase i, respectively. In Equation (8), $S_{q}$ represents the surface where the heat source is applied; similarly, $S_{\theta }$ in Equation (9) represents the surfaces where the convective and radiative boundary conditions are applied. $T_{0}$ is the ambient temperature and $\chi_{1}$ and $\chi_{2}$ are the convective and radiative heat transfer coefficients, respectively.

#### 2.4.1. Phase Fraction Calibration Using CCT-Diagram

The details on $A_{ij}\left(T,\dot{T}\right)$ in Equation (4) and the calibration of the CCT diagram for the phase fraction calculations are provided in this section. In this work, the CCT diagram of EH36 steel presented in [1] was employed for the calibration. The term $A_{ij}\left(T,\dot{T}\right)$ describes the transformation of phase *i* to phase *j* ($A_{ij}$ > 0) or vice versa ($A_{ij}$ < 0). The transformations can be further elaborated by the following equation:

(10) $$A_{ij}=\{\begin{array}{l} k_{ij}\left(T\right)p_{i}-l_{ij}\left(T\right)p_{j}\\ \mathrm{if}\;k_{ij}\left(T\right)p_{i}-l_{ij}\left(T\right)p_{j}>0(i\to j\;\mathrm{transf}.)\\ -k_{ji}\left(T\right)p_{j}+l_{ji}\left(T\right)p_{i}\\ \mathrm{if}\;k_{ji}\left(T\right)p_{j}-l_{ji}\left(T\right)p_{i}>0(j\to i\;\mathrm{transf}.)\\ 0\mathrm{if}\;k_{ij}\left(T\right)p_{i}-l_{ij}\left(T\right)p_{j}\leq 0\\ \mathrm{and}\;k_{ji}\left(T\right)p_{j}-l_{ji}\left(T\right)p_{i}\leq 0\\ \left(\mathrm{no}\;\mathrm{transformation}\;\mathrm{between}\;\mathrm{phases}\;i\;\mathrm{and}\;j\right) \end{array}$$

where $k_{ij}$ and $l_{ij}$ are metallurgical parameters to be determined from the calibration with the CCT-diagram. As Leblond and Devaux suggested [18], the parameters are defined as follows:

(11) $$k_{ij}=\frac{P_{\mathrm{eq}}\left(T\right)}{\tau },l_{ij}=\frac{1-P_{\mathrm{eq}}\left(T\right)}{\tau }$$

Here, $P_{\mathrm{eq}}\left(T\right)$ is known as the equilibrium proportion which is reached at a constant temperature after an infinitely long time and has a value between 0 and 1. While τ indicates the characteristic time necessary for the phase to reach an equilibrium state. The value of $P_{\mathrm{eq}}\left(T\right)$ is determined by considering the start and finishing temperatures ($T_{s}$ and $T_{f}$) of ferritic transformations and is defined as:

(12) $$P_{\mathrm{eq}}\left(T\right)=\frac{T_{s}-T}{T_{s}-T_{f}}$$

The time derivative of the phase may be defined as Equation (13).

(13) $$\frac{dP}{dt}=\frac{P_{\mathrm{eq}}-P}{\tau }$$

**Figure 3:**
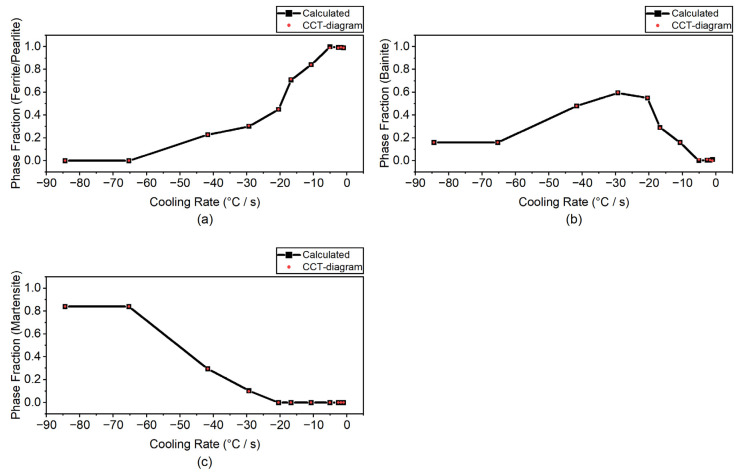
Comparison of the phase fraction calculations using the calibrated parameters in the present study with the ones in the CCT-diagram for (**a**) ferrite/pearlite (**b**) bainite, and (**c**) martensite.

By applying Taylor’s expansion to Equation (13) we can obtain the phases ($P_{n+1}$) at the time $t_{n+1}$ as in Equation (14):

(14) $$P_{n+1}=P_{n}+{\frac{dP}{dt}|}_{n}{\left(\frac{dT}{dt}\right)}^{-1}\Delta T=P_{n}+\frac{P_{\mathrm{eq}}-P}{\tau }{\left(\frac{dT}{dt}\right)}^{-1}\Delta T$$

where $\frac{dT}{dt}$ is the cooling rate while ΔT denotes the temperature difference between $t_{n+1}$ and $t_{n}$. Iterative computations were conducted for the calibration with the CCT curves by finding τ such that the temperature *T_f_* was in agreement with the one from the CCT diagram. For verification of the calibrated metallurgical parameters, the present phase fraction calculations are compared with those in the CCT diagram and plotted in Figure 3 for different cooling rates from −0.98 °C/s to −84.4 °C/s. As shown, the calculated values of the phase fraction are in good agreement with these in the CCT-diagram for different cooling rates considered. 

### 2.5. Mechanical Analysis

The thermo-elastoplastic constitutive model including the transformation plasticity proposed by Leblond et al. [20,21] were used in the present study. In their works, they categorized the multiple phases in steel into two phases: the weak phase (*γ*-phase: the austenitic phase) and the hard phase (*α*-phase: ferrite, bainite, and martensite). Hereafter they are referred as subscript 1 and subscript 2, respectively.

In this work, it was assumed to be an isotropic material behavior for simplicity. The total strain $\varepsilon^{tot}$ is composed of the elastic strain $\varepsilon^{el}$, the thermo-metallurgical strain $\varepsilon^{thm}$, the strain due to transformation plasticity $\varepsilon^{tp}$ and, the strain due to conventional plasticity $\varepsilon^{cp}$, as shown in Equation (15).

(15) $$\varepsilon^{tot}=\varepsilon^{el}+\varepsilon^{thm}+\varepsilon^{tp}+\varepsilon^{cp}$$

where $\varepsilon^{thm}$ is given by:

(16) $$\varepsilon^{thm}=\left(1-z\right)\varepsilon_{1}^{th}\left(T\right)+z\varepsilon_{2}^{th}\left(T\right)$$

Equation (16) describes the volume change induced by phase transformations and thermal dilatation. $\varepsilon_{1}^{th}$ and $\varepsilon_{2}^{th}$ are the thermal strains of the weak and the hard phases, respectively. Such strains are calculated by taking the secant value of temperature-dependent values of the thermal expansion coefficient. z is the phase fraction of the hard phase (α-phase). By using the law of linear weights, the average yield strength of individual phases can be summed together to find the yield strength of the α-phase ($\sigma_{2}^{y}$) as shown in Equation (17):

(17) $$\sigma_{2}^{y}\left(T\right)=\sum_{i}p_{i}\sigma_{i}^{y}$$

where $p_{i}$ denotes the portion of each phase, while $\sigma_{i}^{y}$ is the yield strength of each phase i. The nonlinear mixture’s rule was used to determine the yield stress of all phases and is given by:

(18) $$\sigma^{y}\left(\varepsilon_{1}^{\mathrm{eff}},\varepsilon_{2}^{\mathrm{eff}},T\right)=\left[1-f\left(z\right)\right]\sigma_{1}^{y}\left(T,\varepsilon_{1}^{\mathrm{eff}}\right)+f\left(z\right)\sigma_{2}^{y}\left(T,\varepsilon_{2}^{\mathrm{eff}}\right)$$

Here, $\sigma_{1}^{y}$ and $\sigma_{2}^{y}$, is the yield stress of the weak phase and the hard phase, respectively.

f(z) is the modification factor for the nonlinear mixture rule (See Table 2 in [1]) Leblond’s model characterizes the flow rule for two different regimes, based on whether the equivalent macroscopic stress $\bar{\sigma }$ reaches the phase mixture’s yield stress (for more details see reference [22,23]).

Case 1: transformation plasticity ($\bar{\sigma }\leq \sigma^{y}$)

(19) $${\dot{\varepsilon }}^{p}=\frac{3}{2}\frac{{\dot{\bar{\varepsilon }}}^{p}}{\bar{\sigma }}s=\sqrt{\frac{3}{2}}n\;\mathrm{with}\;n=\sqrt{\frac{3}{2}}\frac{s}{\bar{\sigma }}$$

(20) $${\dot{\bar{\varepsilon }}}^{p}=\sqrt{\frac{3}{2}}\frac{1-z}{\sigma_{1}^{y}\left({\bar{\varepsilon }}_{1}^{\mathrm{eff}}\right)}{\dot{\varepsilon }}_{1}^{eff}∥s∥$$

(21) $${\dot{{\bar{\varepsilon }}_{1}}}^{eff}=\frac{-2\Delta \varepsilon_{1\to 2}^{th}}{1-z}\left(\ln z\right)\dot{z}\hat{h}\left(∥s∥,{\dot{\varepsilon }}_{1}^{eff},{\dot{\varepsilon }}_{2}^{eff}\right)+\sqrt{\frac{3}{2}}\frac{g\left(z\right)}{E}∥s∥+\frac{2\left(\alpha_{1}-\alpha_{2}\right)z\ln z}{1-z}\dot{T}$$

(22) $${\dot{\bar{\varepsilon }}}_{2}^{eff}=-\frac{\dot{z}}{z}{\bar{\varepsilon }}_{2}^{eff}+\omega^{\dot{z}}{\bar{\varepsilon }}_{1}^{eff}$$

(23) $$\bar{\sigma }=\sqrt{\frac{3}{2}s:s}$$

where $\Delta \varepsilon_{1\to 2}^{th}$ is the difference in thermal strain between the weak and hard phase, s is the deviatoric stress, $\hat{h}\left(∥s∥,{\dot{\varepsilon }}_{1}^{eff},{\dot{\varepsilon }}_{2}^{eff}\right)$ is a correction function that accounts for nonlinearities in the stress, g(z) (See Table 3 in [1])) is a modification function for 1/*z* accounting for small values of *z*; E is the Young’s modulus; $\alpha_{1}$ and $\alpha_{2}$ are the thermal expansion coefficients of the two phases; ω is a parameter that takes the value of 0 for all other phases and 1 for martensite and is used to memorize the history of the evolution (refer to [23] for more details). 

Case 2: macroscopic plasticity ($\bar{\sigma }=\sigma^{y}$)

(24) $${\dot{\varepsilon }}^{p}=\frac{3}{2}\frac{{\dot{\bar{\varepsilon }}}^{p}}{\bar{\sigma }}s$$

(25) $$\bar{\sigma }=\sigma^{y}$$

(26) $${\dot{\bar{\varepsilon }}}_{1}^{eff}={\dot{\bar{\varepsilon }}}^{p}$$

(27) $${\dot{\bar{\varepsilon }}}_{2}^{eff}={\dot{\bar{\varepsilon }}}_{2}^{p}-\frac{\dot{z}}{z}{\bar{\varepsilon }}_{2}^{eff}+\omega \frac{\dot{z}}{z}{\bar{\varepsilon }}_{1}^{eff}$$

As shown in Equation (18), $\sigma^{y}$ depends on ${\dot{\bar{\varepsilon }}}_{1}^{eff}$, ${\dot{\bar{\varepsilon }}}_{2}^{eff}$, and T. 

In this work, the stress-update is carried out by employing Hypoelastic formulation. First, it is assumed that material undergoes elastic deformation(i.e., $\Delta \varepsilon^{pl}$ = 0). Hence, the trial stress of the elastic predictor is expressed as follows:

(28) $$\sigma_{n+1}^{\mathrm{trial}}=\sigma_{n}+C^{el}:\Delta {\hat{\varepsilon }}^{\mathrm{RN}}+\Delta C:\varepsilon_{n}^{el}$$

where $\sigma_{n+1}^{\mathrm{trial}}$ is the trial stress at the current time step; $C^{el}$, $\Delta {\hat{\varepsilon }}^{\mathrm{RN}}$, $\varepsilon_{n}^{el}$ are the elastic tangent moduli tensor, the total rotation-neutralized strain increment, and the elastic strain at the previous time step, respectively. In the stress-update process, the trial stress in Equation (28) is calculated first, and then the numerical model determines if the material at the current time step undergoes elastic or plastic deformation by comparison of the trial stress with the yield strength. In case of elastic deformation, the final stress is updated with the trial stress in Equation (28) and the algorithm moves to the next time step. 

**Figure 4:**
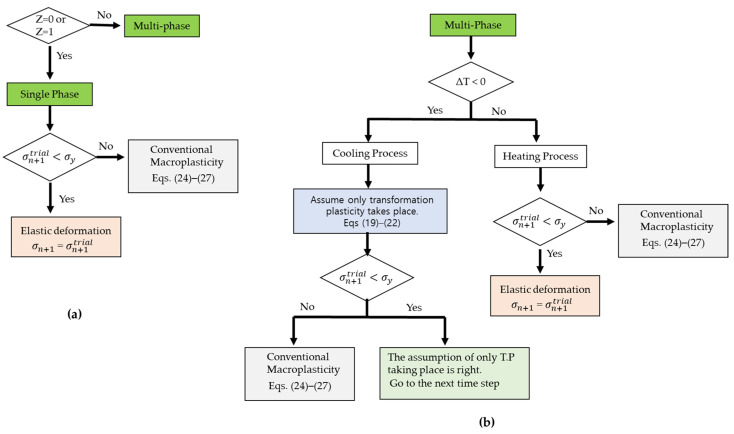
The stress-update process of the present numerical model. (**a**) single phase case; (**b**) multiple phase case.

The plastic deformation is divided into two categories as described above: One is the transformation plasticity ($\bar{\sigma }\leq \sigma^{y}$, occurs in cooling process); the other is the macroscopic plasticity ($\bar{\sigma }=\sigma^{y}$). If the material undergoes the plastic deformation, the plastic relaxation effects are calculated by using the radial-return mapping in a different way depending on whether the type of the plastic deformation is the transformation plasticity (use Equations (19)–(23)) or the conventional macroscopic plasticity (use Equations (24)–(27)). Such plastic relaxation is finally implemented in stress-update to satisfy the final stress state satisfy the yield criterion. More details on the stress-update process is described in the author’s previous works [1]. In addition, the flowchart explaining such process is shown in Figure 4.

## 3. Results and Discussion

The results are analyzed based on the temperature history of the present simulations on the WAAM process. we first look at how the temperature history varies across the different patterns. Then, a comparison is made based on the influence of this temperature history on phase transformations and residual stresses of different patterns, and a discussion on how these residual stresses affect distortion is made.

### 3.1. Temperature Analysis

**Figure 5:**
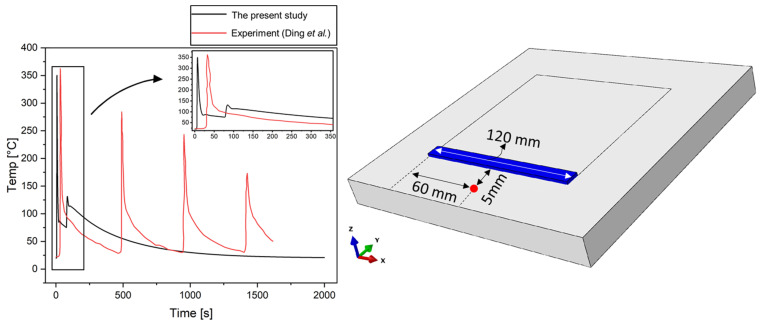
Verification of the current temperature calculations at the point 5 mm away from the deposition path compared with the experimental measurements.

First, the temperature profile is compared with the experimental measurements for verification of the thermal analysis model in the present study. The temperature profile for the alternate case in the present study was selected and compared with the results from Ding et al. [15]. The location of the comparison was selected as the point 5 mm away from the deposition path as in Ding’s case. Figure 5 shows the temperature profile and the location of the comparison. It is worth noting that in this work, the simulations of a single deposition were carried out, while Ding et al. investigated the AM process with multiple depositions. That explains the existence of four peaks on the temperature curve in the case of the experiment, while a single peak temperature is observed in the case of the present study. However, the maximum temperature is similar for both cases. Overall, the maximum temperature and trend of the temperature profile for both cases are in good agreement with each other. The slight discrepancy in the cooling rate may result from the travel speed of the welding torch and/or the boundary conditions.

**Figure 6:**
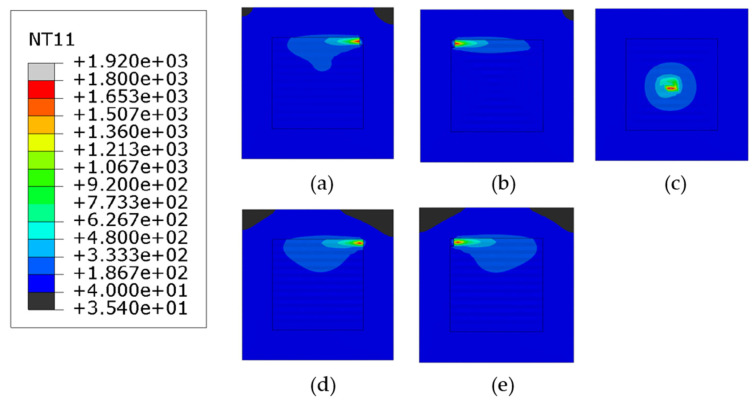
The temperature distributions of all patterns considered at the end of deposition process (**a**) alternate, (**b**) in–out, (**c**) out–in, (**d**) raster, (**e**) zigzag (Top view, unit: °C).

Figure 6 shows the temperature distributions at the end of the deposition. As shown, the scanning pattern does not influence the melt pool temperature. In the case of the out–in spiral scanning, the heat is accumulated at the center of the deposition layer due to its characteristic of the scanning path, and the localized higher temperature is distributed near the center as shown in Figure 6c. Note that the thermal conductivity of steel decreases with the increase of temperature, leading to the reduction of the heat transfer performance. Overall temperature distribution of the substrate and the deposited layer for the out–in case stays between 40–180 °C.

**Figure 7:**
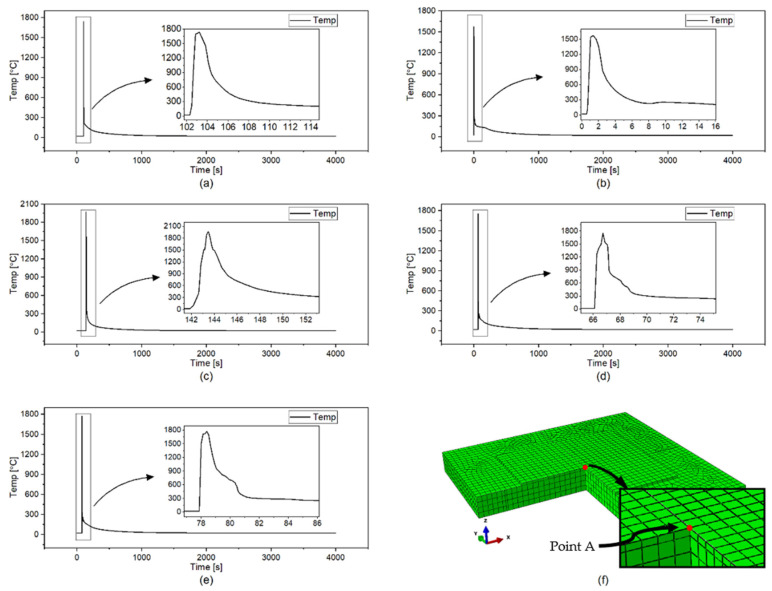
Temperature history at the Point A. (**a**) alternate, (**b**) in–out, (**c**) out–in, (**d**) raster, (**e**) zigzag, (**f**) The location of the Point A.

To further analyze the effect of the scanning pattern on the temperature profile, phase transformation evolution, and residual stress generation, the center point of the deposition layer has been chosen for the investigation as shown in Figure 7f. This point is named hereafter as point A. Figure 7a–e show the temperature history for the alternate, in–out, out–in, raster, and zigzag patterns at Point A. In the case of the alternate scan shown in Figure 7a, the maximum temperature at Point A drops to below 300 °C quicker than any other cases. This is mainly because the distance between each scan pass in the alternate pattern is longer than others, leading to a faster cooling rate. Similar trends are observed in the comparison between the raster and the zigzag cases in Figure 7d,e. The difference between these two scanning patterns is that the raster retains temperatures due to the adjacent passes, while the zigzag is a single continuous pass. Compared with other cases, the out–in case in Figure 7c retains temperature for the longest, due to shorter pattern elements and continual pass. 

### 3.2. Phase Transformation Analysis

In this section, the effect of the scanning pattern on the phase transformation evolution is discussed. Iron atoms in steel are arranged in a certain type of cubic structure, depending on temperature. Ferritic steel (*α*-phase: ferrite/pearlite, bainite, or martensite) has a bcc structure at room temperature, while austenitic (*γ*-phase: austenite) exists above the critical eutectoid temperature (T = 715 °C for EH36 steel [24]). In cooling after the laser deposition, austenitic steel transforms into ferrite, pearlite, bainite, and martensite depending on cooling rate. For high cooling rates, the carbon atoms are trapped into the cell structures with few carbon atoms able to diffuse out of these cells, leading to martensite transformation. On the other hand, for lower cooling rates, the carbon atoms diffuse out of the cell structures and result in ferrite-pearlite, and/or bainite phases depending on the cooling rate [1].

**Figure 8:**
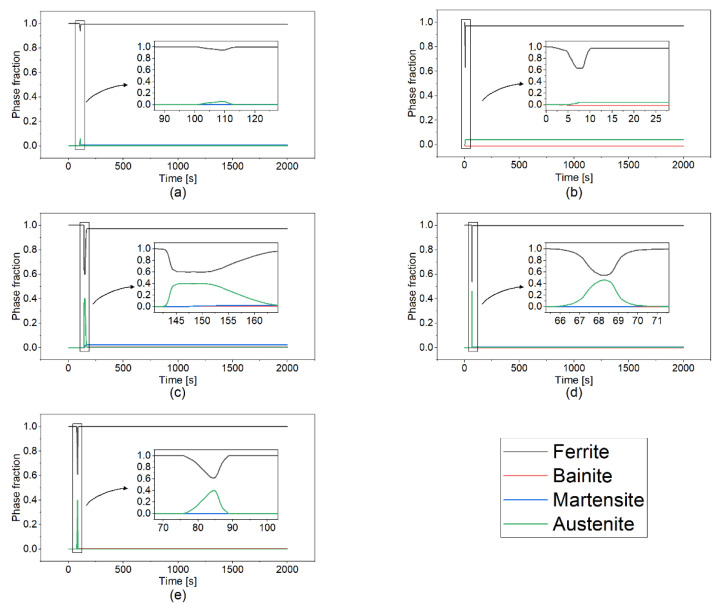
Phase volume fraction at the point A as a function of the scanning pattern: (**a**) alternate, (**b**) in–out, (**c**) out–in, (**d**) raster, and (**e**) zigzag. The location of the point A is explained in the Figure 7f.

Figure 8 shows the phase transformation evolution at the point A for all scanning patterns considered. In the heating cycle, once the temperature passes to the eutectoid temperature, the austenite grains start to be formed and grow. Analysis of the results in Figure 7 and Figure 8 suggests that the time when the temperature reaches the eutectoid temperature (Figure 7) (i.e., T = 715 °C) during the heating cycle is the same with the ones when the austenite phases are started to be formed in Figure 8 for all cases considered in this work. For example, as shown in Figure 7, the temperature reaches the eutectoid temperature in the heating cycle at *t* = 103 s, 2 s, 143 s, 67 s, and 78.5 s in case of the alternate, in–out, out–in, raster, and zigzag scanning, respectively. Such values of the time are in quite good agreement with the ones at which the austenitic transformation starts in Figure 8. In the alternate, raster, and zigzag cases, the ferrite is the dominant phase at the end of the deposition, while in the case of the in–out and out–in patterns, a fraction of martensite and austenite phases can be observed. The formation of bainite is only observed in the out–in case. It is worth noting that the time periods when the temperature is in the range between 750 °C and 300 °C in Figure 7 matches the time windows of the austenitic transformation in Figure 8 in which the austenite phase starts to nucleate and grow. For all the scan patterns considered in this work, the phase volume fraction of ferrite/pearlite at the end of deposition is calculated to be greater than 97%. In the case of the in–out and out–in scan in Figure 8b,c, the phase volume fractions of the martensite are calculated as 3%, and 2%, respectively. Such major formation of pearlite at the end of the layer deposition suggests that air-cooling heat treatment in the present simulations is not fast enough to nucleate the martensite. The major two factors that lead to the martensitic transformation are the high cooling rate and high carbon content in steel [24]. EH36 steel is low-carbon low-alloy steel. In addition, microscopic analysis on the as-printed WAAM ship plate performed by Vahedi et al. [24] also shows that applying the air-cooling heat treatment results in the formation of a ferritic-pearlitic microstructure, homogeneously developed over the entire sample, which is consistent with the present study.

For each case in Figure 8, the results show a different transformation, since it is directly dependent on the temperature history and the cooling rate. The cooling rate differs due to the difference in length of the pattern elements and the number of passes (i.e., starts and stops). 

### 3.3. Stress Analysis

**Figure 9:**
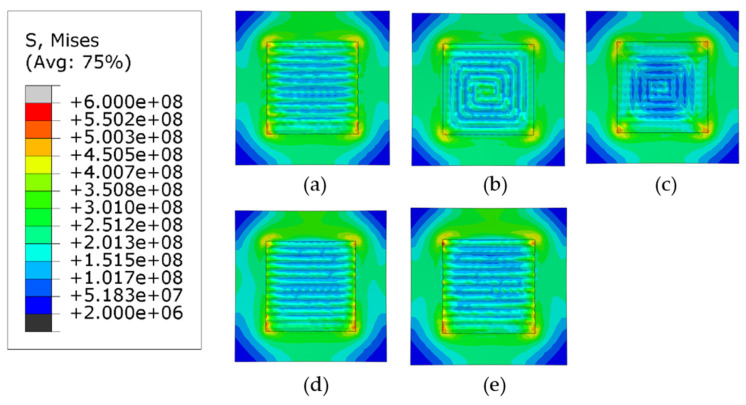
Mises stress distribution of different deposition patterns at the end of the simulation, when phase transformations are not considered, (top view, unit: Pa); (**a**) alternate, (**b**) in–out, (**c**) out–in, (**d**) raster, (**e**) zigzag.

**Figure 10:**
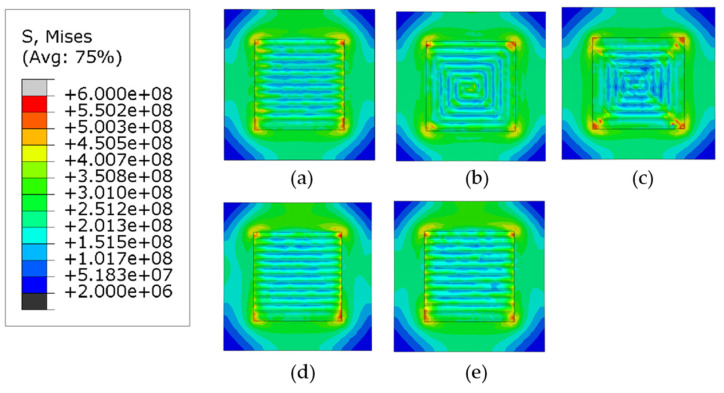
Mises stress distribution of different deposition patterns at the end of the simulation, when phase transformations are considered, (top view, unit: Pa); (**a**) alternate, (**b**) in–out, (**c**) out–in, (**d**) raster, (**e**) zigzag.

Figure 9 and Figure 10 show the Mises stress distribution after the clamps are removed. Figure 9 shows the case when phase transformations have not been considered (herein referred to as Case 1) and Figure 10 shows the contour plots when the phase transformations were considered (herein referred to as Case 2).

Regardless of the phase transformations, the general trend for the mises stresses is that the highest stresses are concentrated at the corners of the deposition area and the lowest values are located at the corners of the substrate. The first trend is observed because the cooling rate at the edge of the deposition area is higher than the internal part of the deposited layer. The other trend is observed because the corners of the substrate are much further away from the deposition area and the difference in temperatures during the deposition of the layers is much higher than the center. For all patterns considered, higher values of stresses are located on the deposition lines which follow the scan vector, whereas lower values are located between them. 

**Figure 11:**
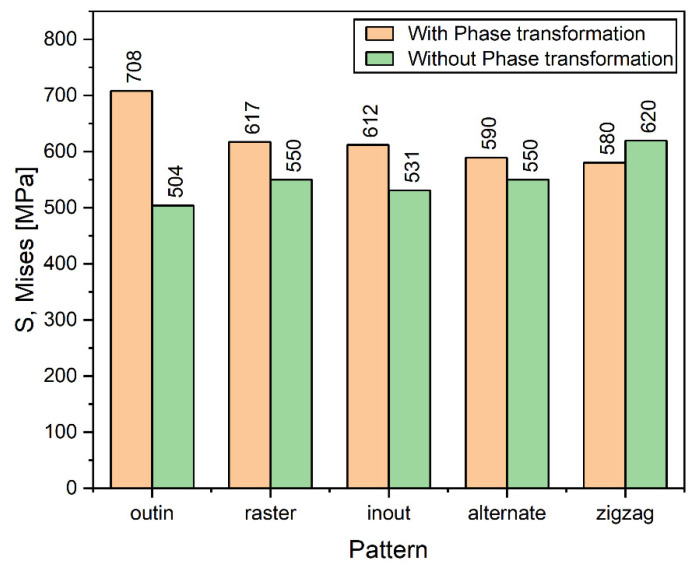
Comparison of maximum Mises stress of different deposition patterns at the end of the simulation.

**Figure 12:**
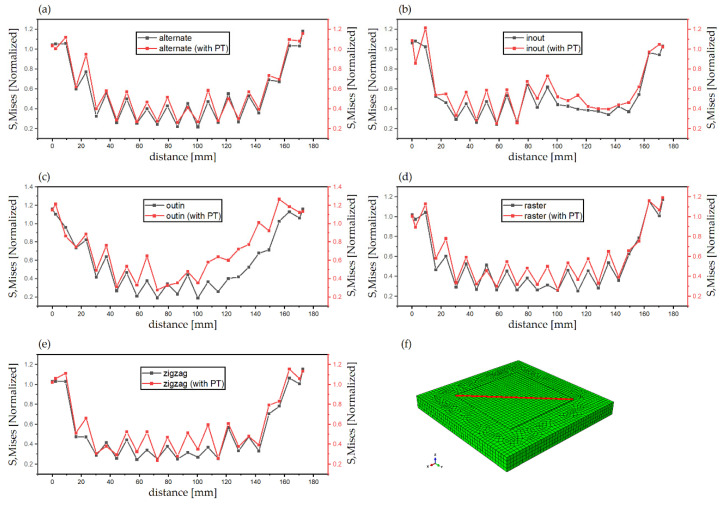
Comparison of the normalized Mises stress (with and without phase transformations (PT)) across diagonal direction of different patterns. (**a**) alternate (**b**) in–out (**c**) out–in (**d**) raster (**e**) zigzag, (**f**) specified diagonal direction.

Figure 11 compares the maximum values of Mises stresses in Cases 1 and 2. For the discontinuous patterns (the alternate and the raster), such difference of the maximum stresses is approximately 50 MPa and Case 2 has higher stresses following the general trend. Similarly, for the continuous contour patterns (the out–in and the in–out), although the same general trend is followed, the difference in stresses for the out–in pattern is nearly 200 MPa, which is much higher than in other cases. The explanation for this deviation is that the heat accumulated at the center of the deposition layer causes the formation of competing phases which significantly affect the levels of stress due to different thermal expansion values, whereas Case 1 can be considered to have just a single phase. The Zigzag pattern is an exception in Case 1 because, although it is a line-type pattern, it is also continuous; therefore, it does not get any cooling time during subsequent passes like the other line-type patterns and is thus an outlier. However, when the phase transformation is considered in case of the zigzag there is a stark difference in the highest value of stresses, it is plausible that due to the stresses now being considered because of the presence of the different phases, it gives a better representation of the overall stresses generated in the part. Figure 12a–e show the comparison of normalized Mises stress distribution across the deposited layer at the end of the simulation for all patterns; the mises stresses have been normalized by the yield stress at room temperature. Both Case 1 and Case 2 are plotted against each other. The plots show that stresses for Case 2 are typically higher for all the patterns and the difference between them is fairly negligible; however, inconsistencies are observed in the out–in and zigzag patterns (Figure 12c,e). This is (as discussed above) due to the presence of thermal gradients due to the nature of continuous patterns. The contour type patterns in Figure 12b,c are similar to each other and are different from the rest of the patterns for the reason that they have shorter pattern elements.

### 3.4. Warpage Analysis

**Figure 13:**
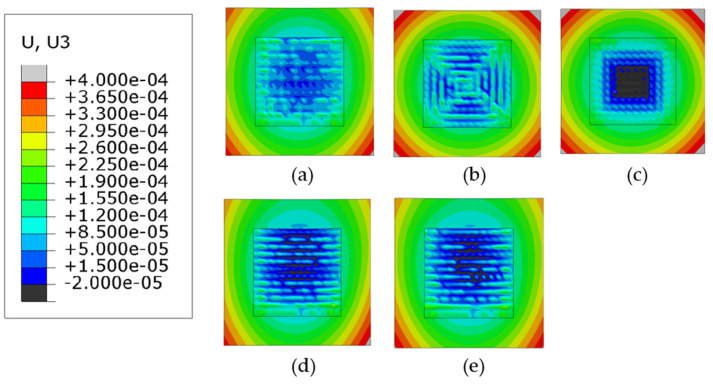
Deformation distribution of different deposition patterns at the end of the simulation, when phase transformations are not considered, (top view, unit: m); (**a**) alternate, (**b**) in–out, (**c**) out–in, (**d**) raster, (**e**) zigzag.

**Figure 14:**
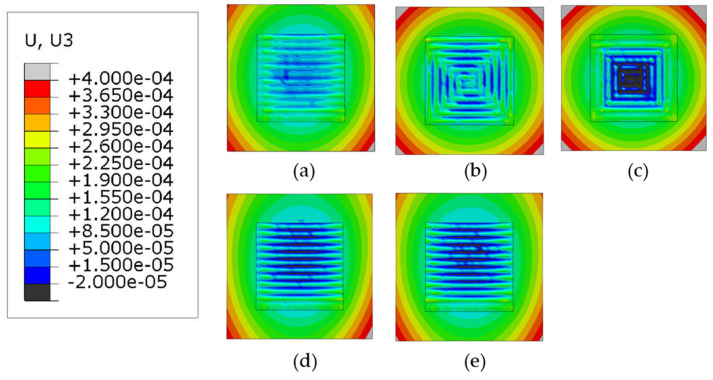
Deformation distribution of different deposition patterns at the end of the simulation, when phase transformations are considered, (top view, unit: m); (**a**) alternate, (**b**) in–out, (**c**) out–in, (**d**) raster, (**e**) zigzag.

Warpage is an important factor to consider in AM processes because it can cause significant distortion in the part after clamp removal [2]. In this study, the build direction is the z-direction; therefore, the contour plots of the displacement in z-direction are analyzed for all patterns as shown in Figure 13 and Figure 14, when phase transformations are not considered (Case 1) and when phase transformations are considered (Case 2), respectively. For all patterns considered, regardless of the two cases, warpage is higher at the start of the deposition process because of the presence of large thermal gradients at the beginning of the deposition process. The deflection in the in–out and the out–in contour plots show the opposite trends. The out–in pattern has the minimum warpage located in the center while the in–out pattern does not. The zigzag and the raster patterns exhibit similar contours for warpage for both Case 1 and Case 2, whereas the alternate pattern is also quite similar to the other line-type deposition patterns. An interesting observation when considering the three-line type patterns (i.e., alternate, raster, and zigzag) is that the deflection in the substrate is the highest on the bottom corner of the substrate where the deformation process is started; this is most likely due to the presence of higher thermal gradients on one side of the substrate at the beginning of the deposition process, a similar trend can also be observed in Nazim and Ruth’s study (Figure 14 of [25]), where they have shown the experimentally measured warpage of their specimens. For the in–out and out–in patterns, the points of maximum deflection can be observed at all the corners of the substrate, with out–in pattern showing the most homogenous warpage distribution out of all the considered patterns.

**Figure 15:**
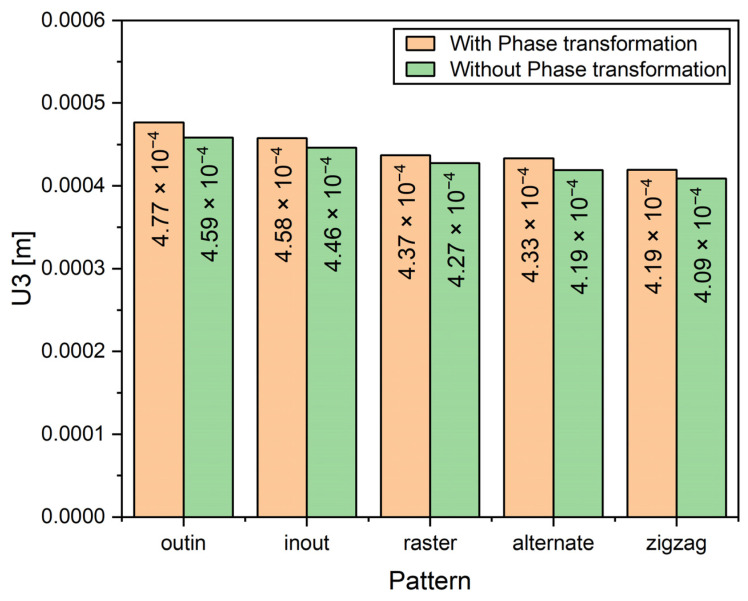
Comparison of maximum deflection of different deposition patterns after clamp removal.

Figure 15 shows the difference between the maximum deflection for all patterns for Cases 1 and 2. Generally, the warpage is higher when phase transformations are considered, on average there is an approximate difference of 0.012 mm between Case 1 and Case 2. Unlike the residual stresses, the general trend is consistent in Case 1 and 2 because the maximum values of warpage are located at the corners of the substrate. When the patterns are considered individually, it is observed that the deflection is highest in the out–in pattern because this pattern has an accumulated negative warpage in the center of the deposition layer, thereby the net effect is that it causes the substrate to warp more at the corners than the other patterns. The three line-type patterns (raster, alternate and zigzag) produce lower warpage than their contour-type counterparts.

## 4. Conclusions

In this study, a sequentially coupled thermo-elasto-plastic FEM with an account of the phase transformations was employed to simulate the residual stresses and warpage of a single layer deposition with five different scan strategies for WAAM. Goldak’s double ellipsoidal heat source model was employed to simulate the heat source. The influence of the phase transformation and transformation plasticity on residual stress and warpage of EH36 steel was investigated for the WAAM fabrication. The layer was simulated using the element activation method. Five scanning strategies were investigated, and the major findings are as follows:

- Discontinuous line-based scanning methods (alternate and raster patterns) provide consistent residual stresses whether phase transformations are considered or not.
- Continuous patterns, specifically out–in and zigzag patterns, show inconsistencies between the residual stresses with phase transformation and non-phase transformation cases, due to continuous heat addition and the higher overall temperature of the substrate. Especially, in the out–in case, the difference in stresses is due to large thermal gradients across the deposited layer.
- Deformation is directly dependent on thermal gradients across the deposition pattern and when phase transformations are considered, the maximum warpage is higher than their non-phase transformed counterpart.
- For accurate simulations of residual stresses and warpage calculations of WAAMed parts when complex patterns are considered for the fabrication, phase transformations should be considered as the patterns directly influence the temperature of the built part and will thus affect the residual stresses and warpage in the part.
- Discontinuous line scanning patterns should be considered wherever possible as they provide the part with uniform residual stress and distortion. An alternate line pattern in this regard is the most consistent overall pattern.

The current study considers a simple geometry with the most commonly used patterns available in literature. It was reported that multiple layer depositions significantly reduce the residual stresses in the subsequent passes [26]. The effects of inter-pass idle times were not taken into consideration in this work. However, the general trend for most low carbon steels can be followed by considering our study for other materials used in WAAM fabrication such as aluminum and Titanium alloys. In order to develop the model with an account of realistic AM conditions such as multi-pass and multi-layer WAAM fabrication, collaborative research is under way. The present study can be extended to study different materials by calibrating the CCT-diagram for the phase transformation of that material. Futhermore, grain information can be incorporated by adding martensitic phase transformations at the grain level by using a phase field formulation [27,28]. Finally, the formulation utilized in the current study can be extended to similar AM processes such as powder bed fusion or electron beam melting [29].

## Figures and Tables

**Table 1:** The parameters for the double ellipsoidal heat source model used in the present study.

*a_f_* (mm)	*a_r_* (mm)	*b* (mm)	*c* (mm)	*f_f_*	*f_r_*	*Q* (W)
2	6	2.5	2.3	0.6	1.4	3000

## Data Availability

Not applicable.
